# Cell-free and intracellular nucleic acids: new non-invasive biomarkers to explore male infertility

**DOI:** 10.1186/s12610-017-0052-0

**Published:** 2017-04-21

**Authors:** Anne Boissière, Anna Gala, Alice Ferrières-Hoa, Tiffany Mullet, Solenne Baillet, Amaël Petiton, Antoine Torre, Samir Hamamah

**Affiliations:** 1 Unité INSERM U1203, Hôpital Saint Eloi, CHRU Montpellier, 80, avenue Fliche, 34295 Montpellier, France; 2 Département de biologie de la reproduction, Hôpital Arnaud de Villeneuve, CHRU Montpellier, 371, avenue du Doyen-Gaston-Giraud, 34295 Montpellier, France

**Keywords:** cf-DNA, cf-mRNA, miRNA, piRNA, Male infertility, Seminal plasma, biomarkers, cf-DNA, cf-mRNA, miRNA, piRNA, Infertilité masculine, Plasma séminal, Biomarqueurs

## Abstract

Male infertility is a devastating problem that affects many couples worldwide. However, the molecular mechanisms and causes of idiopathic male infertility remain unclear. Circulating cell-free nucleic acids have an important role in human physiology and emerging evidence suggests that they play a role in male infertility. This review summarizes recent results on cell-free and intracellular nucleic acids in male infertility and discusses their potential use as biomarkers of male infertility in the clinical practice.

## Résumé

L’infertilité masculine est un problème qui touche de nombreux couples. Cependant, aujourd’hui les mécanismes moléculaires et les causes de l’infertilité masculine idiopathique ne sont pas élucidés. Les acides nucléiques circulant ont un rôle important dans la physiologie et des évidences suggèrent qu’ils jouent un rôle dans l’infertilité masculine. L’objectif de cette revue est de mettre en avant les nouvelles avancées scientifiques sur les acides nucléiques circulant et non-circulant en lien avec l’infertilité masculine et de fournir une vue d’ensemble de leurs utilisation comme biomarqueurs en pratique clinique.

## Background

Cell-free (cf) circulating nucleic acids in human plasma were first described by Mandel et al. in 1948 [1] and their importance as candidate biomarkers was recognized in the early 1990s after the study published by Sorenson et al. [2]. They include cell-free DNAs (cf-DNAs) and cell-free RNAs (cf-RNAs), which comprises messenger RNAs (mRNAs) and three major small non coding RNAs: microRNAs (miRNAs), piwi-interacting RNAs (piRNAs) and small interfering RNAs (siRNAs). Cf-DNAs are DNA fragments that circulate in the bloodstream following their release from apoptotic and/or necrotic cells [3]. They may also be actively secreted by living cells, for instance cancer cells. Therefore, circulating cf-DNA is a non-invasive source of material that can be used to collect genetic and epigenetic information on these cells [4, 5]. Cf-RNAs have been detected in many biological fluids. Like cf-DNA, they can be released by dying cells or be actively secreted by living cells. Therefore, they also may represent promising sources of material for assessing the gene expression profile of cells and tissues. Moreover, it has been demonstrated that extracellular small non coding RNAs may function as a signaling molecules in cell-cell communication [6, 7]. In body fluids, circulating cf-RNAs may be associated in protein complexes, such as density lipoproteins [8], with Argonaute 2 proteins [9], with platelets in human plasma [10] or with microvesicles, such as exosomes that may participate in extracellular genetic exchange [6, 11, 12].

Circulating cell-free nucleic acids are found in and can be easily extracted from different biological fluids [12–14], such as serum or plasma [15], urine [16], seminal plasma [17] and semen [18]. They can be promising biomarkers to detect, monitor and predict the prognosis of several pathologies, particularly cancers, cardiovascular and neurological diseases [19–21]. Moreover, the study of fetal circulating nucleic acids in maternal plasma has opened a new avenue for the development of non-invasive prenatal diagnostic tools, as described by Lo et al. in 1997 [22]. In gynecological and obstetrical disorders, circulating cell free nucleic acids are considered to be potential biomarkers of female infertility [23, 24]. For instance, they can be used to detect ovarian reserve disorders, such as polycystic ovary syndrome [25], and to evaluate the quality of the follicular microenvironment [26].

Infertility is defined as the inability to achieve a clinical pregnancy after one year of regular and unprotected sexual intercourse. Worldwide, it affects approximately 15% of couples in age to procreate and trying to conceive [27, 28]. Female- and male-related causes explain each about one third of all infertility cases. The last third is due to both female and male causes or to no clear cause [29].

Reduced male fertility can be the result of congenital and acquired urogenital abnormalities, infections of the male accessory glands, increased scrotal temperature (varicocele), endocrine disturbances, genetic abnormalities (including chromosomal aberrations, polymorphisms, single or multiple gene mutations), mitochondrial dysfunction, or immunological factors and can be idiopathic in about 40% of patients [30–32].

In the last case, male infertility is defined as idiopathic when no casual factor is found and only semen analysis shows abnormalities, such as no detectable spermatozoa (azoospermia), decreased number of spermatozoa (oligozoospermia), decreased motility (asthenozoospermia) or abnormal morphology (teratozoospermia). When the last three abnormalities occur simultaneously, the syndrome can be described as moderate or severe oligoasthenoteratozoospermia [30, 32]. Epigenetic alterations should also be considered among the idiopathic male infertility etiologies because hyper- and hypo-methylation of imprinted and non-imprinted genes in spermatozoa have been associated with oligo-, astheno-, and/or teratozoospermia [33].

In clinical andrology and in assisted reproductive technology (ART), to document male infertility a detailed medical history should be obtained followed by a through clinical examination, semen analysis, endocrine and genetic evaluation and even testicular biopsy or puncture, to obtain an accurate diagnosis [31]. Most of the tests mentioned above are routinely used in the clinical practice however, these investigations mostly fail to elucidate the cause of male factor infertility. Moreover, these tests are cost- and labour-intensive (particularly, genetic and endocrinology tests), challenging and invasive (biopsy). For example, semen analysis alone is not reliable enough for accurate diagnosis and semen abnormalities do not exclude the possibility of normal fertility [34]. In addition, to diagnose the type of azoospermia a testicular biopsy is carried out to determine whether azoospermia is caused by a blockage (Obstructive Azoospermia, OA) or by primary testicular failure and germ cell loss (Non-Obstructive Azoospermia, NOA). These practices present a number of limitations and non-invasive biomarkers to determine the level of residual spermatogenesis in subfertile patients are necessary. On the other hand, as the efficiency of the existing hormonal treatements is rather low [32, 35], the identification of genetic factors that affect male infertility could provide valuable information for the development of targeted treatments. They could also help determining the causes of idiopathic infertility. In consequence, new non-invasive biomakers and more reliable diagnostics tools are required for discriminating among the different causes of infertility and/or for the diagnosis of subfertility in the clinical practice.

The aim of this review is to summarize the most recent scientific advance concerning cf-DNA, and circulating and intracellular small non-coding RNA and their potential use as non-invasive biomarkers.

## Circulating DNA and male infertility

Cell-free DNA has been detected in human semen [17, 18]. Interestingly, its concentration in semen is much higher than in other body fluids [17]. As reported by Chou et al., cell-free seminal DNA (cfs-DNA) is associated with sperm parameters linked to normal sperm function such as velocity or morphology [18]. cfs-DNA level has been shown to be higher in seminal plasma of azoospermic than normozoospermic patients [17]. These observations suggest that cfs-DNA could be used in a search for biomarkers of sperm quality. On the other hand, it has been hypothesized that epigenetic alterations could cause male infertility..The presence of epigenetic information has been shown on cell-free seminal DNA [36]. This epigenetic information should reflect testicular epigenetic aberrations as semen is a mixture of secretions from the two testes, epididymes, seminal vesicles, bulbourethral glands and prostate [37]. Indeed, Wu et al. showed the existence of a correlation between methylation of specific promoters (such as CCNA1 and DMRT1) in cell-free seminal DNA and sperm physiopathology. In this study, the cfs-DNA methylation of these promoters was shown to be higher in the hypospermatogenesis group than in other groups with spermatogenic defects and normozoospermic patients [38]. These findings indicate that cell-free seminal DNA contains the epigenetic information of the male genital tract and could be a novel, non-invasive biomarker to detect spermatogenesis abnormalities.

## Circulating and intracellular RNAs in male infertility

Besides cell-free DNA detection, cell-free RNA analysis presents theoretical advantages since it can provide valuable information on the gene expression patterns. In human semen, cf-RNA is a mix of transcripts derived from the male reproductive organs. It is fairly stable, particle-associated and more concentrated than in any other biological fluid [39, 40].

In this chapter, we will summarize the recent findings on cell-free mRNA, circulating and non-circulating small non-coding RNA related to male infertility.

### Cell-free mRNA in male infertility

A recent integrated analysis of both mRNA and miRNA profiles in testis samples showed that many mRNAs and miRNAs are differentially expressed in patients with non-obstructive azoospermia (NOA) compared with patients with obstructive azoospermia (OA) or with controls (normozoospermia) [41]. This suggests that the two azoospermia forms (OA and NOA) may be distinguished at the transcriptional level. Similarly, studies on the potential of cell-free seminal mRNAs as new non-invasive biomarkers have shown that they could be used for the diagnosis of male infertility. For instance, in seminal plasma of NOA patients, the detection of the cell-free mRNA *DDX4* transcript (a germ cell-specific marker) was highly dependent on the presence of germ cells in testis. In contrast, the absence of the germ cell-specific (DDX4) RNA in cell-free seminal RNA was consistent with the histologic diagnostic of obstructive azoospermia. This finding suggests that cfs-mRNA DDX4 might be used to evaluate the type of azoospermia [42]. In addition, Pansa et al. showed that the germ cell-specific *ESX1* transcript is down-regulated in seminal plasma of patients with NOA compared with controls (normozoospermia). Moreover, they found that the change in expression is correlated with the severity of the spermatogenic defects [43]. Consequently, both *DDX4* and *ESX1* germ cell-specific mRNAs could be used as suitable molecular markers to predict the presence of residual spermatogenesis.

In conclusion, semen cf-RNA seems to be a promising non-invasive biomarker of the male reproductive system for diagnostic medicine and research due to its abundance, stability and the information it carries about the reproductive tract organs [40, 44].

### MicroRNAs in male infertility

MiRNAs play a crucial role in numerous aspects of cell biology, such as cell differentiation, metabolism and apoptosis. They are small (about 22 nucleotide-long), non-coding RNAs that are evolutionarily conserved and that modulate gene expression post-transcriptionally by targeting specific mRNAs [45]. It has been shown that miRNAs can affect mRNA stability and in some cases influence protein synthesis through target cleavage and/or translation inhibition [45]. Human sperm contain a stable population of miRNAs related to embryogenesis [40] and to spermatogenesis [46, 47]. Their role in the control of male germ cell differentiation [48] highlights the fundamental contribution of these molecules to fertility regulation.

Salas-Huetos et al*.* listed 221 miRNAs that are consistently detected in spermatozoa of fertile men. Among the ten most expressed miRNAs, three directly control spermatogenesis because they are involved in the regulation of the E2F-pRB pathway during male meiosis (hsa-miR-34b-3p), in cell cycle progression (hsa-miR-132-3p) and in sperm differentiation (hsa-miR-191-5p). The others are linked to cancer and aging (hsa-miR-30b-5p, hsa-miR-30c-5p, hsa-miR-375, hsa-miR-19b-3p, hsa-miR-200c-3p), or to unknown biological functions (hsa-miR-891a, hsa-miR-1233-3p) (see Table 1) [46].

**Table 1:** List of miRNAs related to male idiopathic fertility disorders and their putative functions

Patients	Samples	miRNA expression	hsa-miRNA	Putative functions
Fertile (controls)	Spermatozoa	-	miR-34b-3p [46]	E2F-pRb regulation
		-	miR-132-3p [46]	Cell cycle progression
		-	miR-191-3p [46]	Sperm differentiation
		-	miR-30b-5p, miR-30c-5p, miR-375, miR-19b-3p, miR-200c-3p [46]	Cancer and aging
		-	miR-891a, miR-1233-3p [46]	Unknown biological function
NOA	Testis tissue	Down	miR-1, miR-181a, miR-221, miR-9*, miR-371, miR-372, miR-373 [49]	Development of human testis germ cell tumors
Idiopathic infertility (NOA, Asthenozoospermia, Oligozoospermia, Astheno-Oligozoospermia)	Seminal plasma, Spermatozoa, Extracellular Microvesicles,	Up	mir-429 [36, 48, 51, 52]	Spermatogenesis
			miR-1275 [47, 53]	
	Spermatozoa, Extracellular Microvesicles	Down	miR-15b [47, 48, 51, 53]	
	Spermatozoa, Seminal plasma	Up	miR-141 [36, 48, 51]	Embryonic development
	Spermatozoa	Up	miR-19b [47, 54], miR-483-5p [47, 53]	Unknown
	Spermatozoa	Down	miR-19a [48, 53], miR-1973 [48, 51]	Unknown
	Spermatozoa, Extracellular Microvesicles		miR-28-5p [47, 53]	
NOA	Seminal plasma, Spermatozoa, Testis tissue	Up	miR-429 [36, 48, 52]	Spermatogenesis
	Spermatozoa	Down	miR-34* [52], miR-34c-5p [48, 52, 55], miR-122 [48, 52, 55]	
Asthenozoospermia	Spermatozoa	Up	miR-27a [47, 57]	Signaling pathway
	Spermatozoa	Up	miR-548b-5p, miR548c-5p, miR-548d-5p [47]	
	Spermatozoa	Down	miR-34b-3p [47, 51]	Spermatogenesis
	Spermatozoa	Down	miR-520 h, miR-520d-3p [47]	Embryonic development
Oligo-Astheno-Zoospermia	Spermatozoa	Down	miR-15a, miR-15b, miR-30, miR-34b, miR-34c-5p, miR-193-5p [51, 53]	Spermatogenesis
Oligozoospermia and Teratozoospermia	Spermatozoa	Down	miR-151-5p [47]	Aging
			miR-935, miR-125a-3p [47]	Cell cycle and chromatin modification
			miR-132-5p, miR-320b, miR-195-5p [47]	Cellular component morphogenesis and cell morphogenesis

Some studies reported that the expression profiles of specific miRNA populations from tissues, cells and fluids are altered in males with different fertility problems (Table 1). Specifically, the first report showed that the testis miRNA expression pattern of patients with NOA is different from that of fertile males. The authors could identify 154 miRNAs that are down-regulated and 19 that are up-regulated in testis samples from patients with NOA compared with controls [49]. Among these differentially expressed miRNAs, there are several members of the hsa-miR-17-92 cluster (hsa-miR-1, hsa-miR-181a, hsa-miR-221 and hsa-miR-9*) and of the hsa-miR-371,2,3 cluster (hsa-miR-371, hsa-miR-372, hsa-miR-373) (see Table 1). MiRNAs from both clusters are downregulated in patients with NOA. Moreover, some of these miRNAs are potential novel oncogenes that might participate in the development of human testicular germ cell tumors ([49] and references therein). Then, Abu-Halima et al. showed that hsa-miR-34b*, hsa-miR-34b, hsa-miR-34c-5p, hsa-miR-449a and hsa-miR-449b, which are structurally similar, are downregulated in testes of infertile men with different histopathologic patterns (the most common feature of male-factor infertility). This suggests that these miRNAs could contribute to regulate male germ and somatic cell development and that their alterations might be associated with reproductive abnormalities [50].

The study of miRNAs in idiopathic male infertility is complex because the genetic cause cannot be identified in many patients and due to the presence of multiple factors that can influence the phenotype. Here, we will focus on miRNAs that are up- or down-regulated in seminal plasma, spermatozoa or extracellular microvesicles (including exosomes) of infertile patients compared with controls. These results are representative of the findings reported by studies on male subfertility in the last years (Table 1). To be considered as representative, a miRNA has to be deregulated and show similar expression profiles in at least two different studies and/or by using two different techniques, such as microarray, high-throughput sequencing or RT-qPCR. In summary, six studies on male idiopathic infertility (patients with NOA, asthenozoospermia, oligozoospermia or oligoasthenozoospermia) found that five miRNAs are upregulated and four downregulated. Among these miRNAs, hsa-miR-429 [36, 51, 52] and hsa-miR-1275 [47, 53], which are up-regulated, and hsa-miR-15b [47, 51, 53], which is down-regulated, are involved in spermatogenesis. Moreover, hsa-miR-141 [36, 51], which is involved in embryonic development, is upregulated. Finally, hsa-miR-19b [47, 54] and hsa-miR-483-5p [47, 53], which are upregulated, and hsa-miR-28-5p [47, 53], hsa-miR-19a [48, 53] and hsa-miR-1973 [48, 51], which are downregulated, are expressed in spermatozoa, but they have not been associated with spermatogenesis or related processes.

When focusing on the miRNA differential expression patterns in NOA, three studies pinpointed four miRNAs that are involved in spermatogenesis [36, 52, 55]: hsa-miR-34b*, hsa-miR-34c-5p and hsa-miR-122, which are downregulated [52, 55], and hsa-miR-429, which is upregulated [36, 52].

In patients with asthenozoospermia, two studies found that hsa-miR-27a [47, 56, 57], hsa-miR-548b-5p, hsa-miR-548c-5p and hsa-miR-548d-5p are up-regulated [47], while hsa-miR-34b-3p [47, 51], hsa-miR-520 h and hsa-miR-520d-3p are downregulated [47]. These miRNAs are expressed in spermatozoa and are involved in spermatogenesis (hsa-miR-34b-3p, hsa-miR-27a), embryonic development (hsa-miR-520 family) or in signaling pathways and human tumorigenesis (hsa-miR-548 family). The two last miRNA families have not yet been associated with spermatogenesis.

Comparison of the results of the two studies by Abu-Halima et al. indicates that six miRNAs are downregulated in patients with oligoasthenozoospermia compared with normozoospermic patients. Among them, hsa-miR-15a, hsa-miR-15b, hsa-miR-30, hsa-miR-34b, hsa-miR-34c-5p and hsa-miR-193b-5p have a direct role in spermatogenesis [51, 53].

Salas et al. were the first to perform miRNA profiling in spermatozoa from patients with teratozoospermia (n = 10) and oligozoospermia (n = 10) and to associate these expression patterns with semen parameters. In the teratozoospermic sperm samples, they identified eight downregulated miRNAs and eleven upregulated miRNAs. In the oligozoospermic samples, 15 miRNAs were downregulated and three were upregulated. Overall, six miRNAs were downregulated in both conditions: hsa-miR-151-5p that is related to aging; hsa-miR-935 and hsa-miR-125a-3p that are related to cell cycle and chromatin modification; hsa-miR-132-5p, hsa-miR-320b and hsa-miR-195-5p that are involved in cell and cellular component morphogenesis [47].

### MicroRNAs and male infertility: molecular mechanisms

MiRNAs modulate gene expression post-transcriptionally by targeting specific mRNAs. Several studies showed that some miRNAs mediate the expression of genes that are involved in idiopathic male infertility. For instance, hsa-miR-27a is closely associated with spermatogenesis and infertility. In patients with asthenoteratozoospermia, increased expression of hsa-miR-27a mediates the repression of the Cysteine-RIch Secretory Protein2 (*CRISP2*) gene that plays a role in sperm motility, acrosome reaction and gamete fusion. Spermatozoa lacking CRISP2 exhibit low sperm motility and abnormal morphology [57].

Estrogen receptors (ERs) mediate estrogen action in the regulation of the hypothalamus-pituitary-testis axis and have a key role in male infertility. Dysfunction of this axis impairs spermatogenesis, leading to reduction of sperm density, sperm motility and morphology [58] and seminiferous tubules [59]. In sperm of patients with oligozoospermia, hsa-miR-100 and hsa-let-7b, two miRNAs that target the ER genes, are overexpressed, while ER expression levels are decreased [60]. Moreover, it has been reported that in severe oligozoospermia, hsa-miR-34c-3p downregulates Phosphatidylinositol-Specific Phospholipase C, X Domain containing 3 (*PLCXD3*). The PLCXD3 protein is expressed at later stages of spermatogenesis [61]. Therefore, these miRNAs might have a diagnostic and prognostic value in infertile men with oligozoospermia.

Very recently, Tang et al. showed that hsa-miR-210 is involved in spermatogenesis by targeting Insulin-like Growth Factor 2 (IGF2). IGF2, a component of the insulin/IGF system, regulates spermatogenesis through its generalized effects on cell proliferation, growth, differentiation and survival and also more specifically by stimulating spermatogonial DNA synthesis and Sertoli cell proliferation [62]. Therefore, hsa-miR-210 could be a potential NOA biomarker.

### MicroRNAs as predictive biomarkers in ART

In ART, intra-cytoplasmic sperm injection (ICSI) is the most commonly used IVF procedure overcoming male fertility problems. Recent progress has enabled the recovery of testicular sperm from patients with NOA for ICSI. However, these spermatozoa offer a limited value for ICSI and the outcome is usually poor. Moreover, gene abnormalities, which may affect the developing zygote, are generally not assessed in the current clinical practice. Therefore, biomarkers to better evaluate semen quality are necessary. The most abundant miRNA in human sperm is hsa-miR-34c [63] and it is involved in spermatogenesis. Recently, Cui et al. showed that hsa-miR-34c levels in spermatozoa are correlated with ICSI outcomes [64]. Specifically, the percentage of good quality embryos at day 3 was higher in the group with higher hsa-miR-34c expression in spermatozoa. This finding suggests that hsa-miR-34c could become a predictive biomarker to identify defective spermatozoa.

### Piwi-interacting RNAs in male infertility

Unlike miRNAs which are expressed in a variety of tissues and cells, piwi-interacting RNAs (piRNAs) are expressed mainly in germ cells especially pachytene spermatocytes and spermatids in human testes [65]. piRNAs are longer (about 29 nucleotide-long) than miRNA and form RNA-protein complexes through interactions with PIWI proteins [66]. These piRNA complexes have been linked to both epigenetic and post-transcriptional gene silencing of retrotransposons and other genetic elements in germ line cells, particularly those in spermatogenesis [67–69].

Cell-free piRNAs have been detected in human semen. Interestingly, cell-free seminal piRNAs levels gradually decrease from fertile patients to asthenozoospermia patients and then to azoospermia patients; moreover they were found to be correlated with sperm viability. A piRNA signature of 5 piRNAs (piR-31068, piR-31925, piR-43771, piR-43773 and piR-30198) that distinguishes fertile from infertile patients has been identified. This panel could be used as specific non-invasive biomarker of male infertility [12]. New studies on piRNAs should open up new avenues in research and diagnostic since they can provide firsthand information about gene disorders and dysregulation in the male reproductive system [70, 71].

## Limitations in biomarker identification and validation

To identify and to validate small non-coding RNAs as infertility biomarkers, a complete survey of the literature is crucial.. Indeed, in the literature, a same miRNA can be either up- or down-regulated, leading to misinterpretations. For example, hsa-miR-122 is down-regulated in spermatozoa of patients with NOA [52] or with oligo- and asthenozoospermia [51]. On the other hand, in semen, it is downregulated in patient with NOA, but up-regulated in patients with asthenozoospermia [55]. This could reflect disorder- or tissue/cell-specific variations, but also differences in sample processing. Methodological and technical approaches are generally not uniform between laboratories and this can lead to discordant results. Moreover, as described very recently by Dong et al., misleading factors might exist when measuring seminal cf-RNA levels. For instance, heat exposure or long sexual abstinence increases semen cf-RNA levels [72]. These types of information could represent a new entry to improve the statistical analysis of raw data.

Therefore, to be considered as promising biomarker, a given small non-coding RNA should have the most significant expression difference between healthy controls and patients and between healthy and pathological tissues/cells/fluids. Moreover, it should be assessed in other relevant diseases because a candidate biomarker may have a similar expression profile also in other related disorders. Finally, validation studies should be performed using a large number of clinical samples.

## Conclusions

Considering the number of studies carried out in the field, little has been clarified about circulating nucleic acids in spermatogenesis and in male infertility. Here, we provide a complete summary of the consistent data on circulating nucleic acids and intracellular miRNAs in male infertilty. Altogether, these data suggest that cf-DNAs, cf-mRNAs, miRNAs and piRNAs may be of prognostic and diagnostic value. However, a more detailed description of seminal plasma constituents is required to identify specific biomarkers of male reproductive defects. Moreover, the identification of infertility-related cf-DNAs and miRNAs in blood might provide powerful biomarkers to explore male infertility beyond semen analysis. New avenues of research could be opened for the development of new treatments of male infertility or even for the design of male contraceptive drugs.

## Abbreviations

ART: Assisted reproductive technology
ATZ: Asthenoteratozoospermia
cf-DNA: Cell-free DNA
cf-RNA: Cell-free RNA
CRISP2: Cysteine-rich secretory protein2
hsa: Homo SApiens
ICSI: Intra-cytoplasmic sperm injection
IGF2: Insulin-like growth factor2
miRNA: microRNAs
mRNA: Messenger RNA
NOA: Non-obstructive azoospermia
OA: Obstructive azoospermia
piRNA: Piwi interacting RNA
PLCXD3: Phosphatidylinositol-Specific Phospholipase C, X Domain containing3
siRNA: Small interfering RNA

## References

[1] Mandel P, Metais P (1948). Les acides nucléiques du plasma sanguin chez l’homme. C R Séances Soc Biol Ses Fil.

[2] Sorenson GD, Pribish DM, Valone FH, Memoli VA, Bzik DJ, Yao SL (1994). Soluble normal and mutated DNA sequences from single-copy genes in human blood. Cancer Epidemiol Biomarkers Prev.

[3] Stroun M, Lyautey J, Lederrey C, Olson-Sand A, Anker P (2001). About the possible origin and mechanism of circulating DNA. Clin Chim Acta.

[4] Warton K, Lin V, Navin T, Armstrong NJ, Kaplan W, Ying K (2014). Methylation-capture and next-generation sequencing of free circulating DNA from human plasma. BMC Genomics.

[5] Warton K, Samimi G (2015). Methylation of cell-free circulating DNA in the diagnosis of cancer. Front Mol Biosci.

[6] Valadi H, Ekström K, Bossios A, Sjöstrand M, Lee JJ, Lötvall JO (2007). Exosome-mediated transfer of mRNAs and microRNAs is a novel mechanism of genetic exchange between cells. Nat Cell Biol.

[7] Belleannée C (2015). Extracellular microRNAs from the epididymis as potential mediators of cell-to-cell communication. Asian J Androl.

[8] Vickers KC, Palmisano BT, Shoucri BM, Shamburek RD, Remaley AT (2011). MicroRNAs are transported in plasma and delivered to recipient cells by high-density lipoproteins. Nat Cell Biol.

[9] Arroyo JD, Chevillet JR, Kroh EM, Ruf IK, Pritchard CC, Gibson DF (2011). Argonaute2 complexes carry a population of circulating microRNAs independent of vesicles in human plasma. Proc Natl Acad Sci.

[10] McDonald JS, Milosevic D, Reddi HV, Grebe SK, Algeciras-Schimnich A (2011). Analysis of circulating MicroRNA: preanalytical and analytical challenges. Clin Chem.

[11] Turchinovich A, Samatov TR, Tonevitsky AG, Burwinkel B (2013). Circulating miRNAs: cell–cell communication function?. Front Genet.

[12] Hong Y, Wang C, Fu Z, Liang H, Zhang S, Lu M (2016). Systematic characterization of seminal plasma piRNAs as molecular biomarkers for male infertility. Sci Rep.

[13] Weber JA, Baxter DH, Zhang S, Huang DY, How Huang K, Jen Lee M (2010). The MicroRNA Spectrum in 12 Body Fluids. Clin Chem.

[14] Tzimagiorgis G, Michailidou EZ, Kritis A, Markopoulos AK, Kouidou S (2011). Recovering circulating extracellular or cell-free RNA from bodily fluids. Cancer Epidemiol.

[15] Tsang JCH, Lo YM (2007). Circulating nucleic acids in plasma/serum. Pathol (Phila).

[16] Hoque MO, Begum S, Topaloglu O, Chatterjee A, Rosenbaum E, Van Criekinge W (2006). Quantitation of promoter methylation of multiple genes in urine DNA and bladder cancer detection. J Natl Cancer Inst.

[17] Li H-G, Huang S-Y, Zhou H, Liao A-H, Xiong C-L (2009). Quick recovery and characterization of cell-free DNA in seminal plasma of normozoospermia and azoospermia: implications for non-invasive genetic utilities. Asian J Androl.

[18] Chou JS, Jacobson JD, Patton WC, King A, Chan PJ (2004). Modified Isocratic capillary electrophoresis detection of cell-free DNA in semen. J Assist Reprod Genet.

[19] O’Driscoll L (2007). Extracellular nucleic acids and their potential as diagnostic, prognostic and predictive biomarkers. Anticancer Res.

[20] Swarup V, Rajeswari MR (2007). Circulating (cell-free) nucleic acids - A promising, non-invasive tool for early detection of several human diseases. FEBS Lett.

[21] Mitchell PS, Parkin RK, Kroh EM, Fritz BR, Wyman SK, Pogosova-Agadjanyan EL (2008). Circulating microRNAs as stable blood-based markers for cancer detection. Proc Natl Acad Sci.

[22] Lo YM, Corbetta N, Chamberlain PF, Rai V, Sargent IL, Redman CW (1997). Presence of fetal DNA in maternal plasma and serum. Lancet Lond Engl.

[23] Scalici E, Mullet T, Ferrières Hoa A, Gala A, Loup V, Anahory T (2015). Les acides nucléiques circulants et infertilité. Gynécol Obstétrique Fertil.

[24] Traver S, Assou S, Scalici E, Haouzi D, Al-Edani T, Belloc S (2014). Cell-free nucleic acids as non-invasive biomarkers of gynecological cancers, ovarian, endometrial and obstetric disorders and fetal aneuploidy. Hum Reprod Update.

[25] Scalici E, Traver S, Mullet T, Molinari N, Ferrières A, Brunet C (2016). Circulating microRNAs in follicular fluid, powerful tools to explore in vitro fertilization process. Sci Rep.

[26] Traver S, Scalici E, Mullet T, Molinari N, Vincens C, Anahory T, Hamamah S. Cell-free DNA in Human Follicular Microenvironment: New Prognostic Biomarker to Predict in vitro Fertilization Outcomes. PLoS ONE. 2015;10(8):e0136172. doi:10.1371/journal.pone.0136172. 10.1371/journal.pone.0136172 PMC4545729 26288130

[27] Thoma ME, McLain AC, Louis JF, King RB, Trumble AC, Sundaram R (2013). Prevalence of infertility in the United States as estimated by the current duration approach and a traditional constructed approach. Fertil Steril.

[28] Mascarenhas MN, Flaxman SR, Boerma T, Vanderpoel S, Stevens GA (2012). National, regional, and global trends in infertility prevalence since 1990: a systematic analysis of 277 health surveys. PLoS Med.

[29] CDC [Internet]. Centers for disease control and prevention; 2016. Available from: http://www.cdc.gov/reproductivehealth/infertility

[30] Tarín JJ, García-Pérez MA, Hamatani T, Cano A (2015). Infertility etiologies are genetically and clinically linked with other diseases in single meta-diseases. Reprod Biol Endocrinol.

[31] Esteves SC, Agarwal A (2011). Novel concepts in male infertility. Int Braz J Urol.

[32] Jungwirth A, Giwercman A, Tournaye H, Diemer T, Kopa Z, Dohle G (2012). European association of urology guidelines on male infertility: The 2012 update. Eur Urol.

[33] Boissonnas CC, Jouannet P, Jammes H (2013). Epigenetic disorders and male subfertility. Fertil Steril.

[34] Guzick DS, Overstreet JW, Factor-Litvak P, Brazil CK, Nakajima ST, Coutifaris C (2001). Sperm morphology, motility, and concentration in fertile and infertile men. N Engl J Med.

[35] Esteves SC, Zini A, Aziz N, Alvarez JG, Sabanegh ES, Agarwal A (2012). Critical appraisal of World Health Organization’s new reference values for human semen characteristics and effect on diagnosis and treatment of subfertile men. Urology.

[36] Wu W, Qin Y, Li Z, Dong J, Dai J, Lu C (2013). Genome-wide microRNA expression profiling in idiopathic non-obstructive azoospermia: significant up-regulation of miR-141, miR-429 and miR-7-1-3p. Hum Reprod.

[37] Robert M, Gagnon C (1994). Sperm motility inhibitor from human seminal plasma: presence of a precursor molecule in seminal vesicle fluid and its molecular processing after ejaculation. Int J Androl.

[38] Wu C, Ding X, Tan H, Li H, Xiong C (2016). Alterations of testis-specific promoter methylation in cell-free seminal deoxyribonucleic acid of idiopathic nonobstructive azoospermic men with different testicular phenotypes. Fertil Steril.

[39] Huang S, Li H, Ding X, Xiong C (2009). Presence and characterization of cell-free seminal RNA in healthy individuals: implications for noninvasive disease diagnosis and gene expression studies of the male reproductive system. Clin Chem.

[40] Li H, Huang S, Guo C, Guan H, Xiong C (2012). Cell-free seminal mRNA and MicroRNA exist in different forms. PLoS ONE.

[41] Zhuang X, Li Z, Lin H, Gu L, Lin Q, Lu Z (2015). Integrated miRNA and mRNA expression profiling to identify mRNA targets of dysregulated miRNAs in non-obstructive azoospermia. Sci Rep.

[42] Li H, Wu C, Gu X, Xiong C (2012). A novel application of cell-free seminal mRNA: non-invasive identification of the presence of germ cells or complete obstruction in men with azoospermia. Hum Reprod.

[43] Pansa A, Sirchia SM, Melis S, Giacchetta D, Castiglioni M, Colapietro P (2014). ESX1 mRNA expression in seminal fluid is an indicator of residual spermatogenesis in non-obstructive azoospermic men. Hum Reprod.

[44] Wang L, Lv J, Guo C, Li H, Xiong C (2014). Recovery of cell-free mRNA and microRNA from human semen based on their physical nature. Biotechnol Appl Biochem.

[45] Bartel DP (2004). MicroRNAs: genomics, biogenesis, mechanism, and function. Cell.

[46] Salas-Huetos A, Blanco J, Vidal F, Mercader JM, Garrido N, Anton E (2014). New insights into the expression profile and function of micro-ribonucleic acid in human spermatozoa. Fertil Steril.

[47] Salas-Huetos A, Blanco J, Vidal F, Godo A, Grossmann M, Pons MC (2015). Spermatozoa from patients with seminal alterations exhibit a differential micro-ribonucleic acid profile. Fertil Steril.

[48] Kotaja N (2014). MicroRNAs and spermatogenesis. Fertil Steril.

[49] Lian J, Zhang X, Tian H, Liang N, Wang Y, Liang C (2009). Altered microRNA expression in patients with non-obstructive azoospermia. Reprod Biol Endocrinol RBE.

[50] Abu-Halima M, Backes C, Leidinger P, Keller A, Lubbad AM, Hammadeh M (2014). MicroRNA expression profiles in human testicular tissues of infertile men with different histopathologic patterns. Fertil Steril.

[51] Abu-Halima M, Hammadeh M, Schmitt J, Leidinger P, Keller A, Meese E (2013). Altered microRNA expression profiles of human spermatozoa in patients with different spermatogenic impairments. Fertil Steril.

[52] Abu-Halima M, Hammadeh M, Backes C, Fischer U, Leidinger P, Lubbad AM (2014). Panel of five microRNAs as potential biomarkers for the diagnosis and assessment of male infertility. Fertil Steril.

[53] Abu-Halima M, Ludwig N, Hart M, Leidinger P, Backes C, Keller A (2016). Altered micro-ribonucleic acid expression profiles of extracellular microvesicles in the seminal plasma of patients with oligoasthenozoospermia. Fertil Steril.

[54] Wu W, Hu Z, Qin Y, Dong J, Dai J, Lu C (2012). Seminal plasma microRNAs: potential biomarkers for spermatogenesis status. Mol Hum Reprod.

[55] Wang C, Yang C, Chen X, Yao B, Yang C, Zhu C (2011). Altered profile of seminal plasma MicroRNAs in the molecular diagnosis of male infertility. Clin Chem.

[56] Zhu H, Wu H, Liu X, Evans BR, Medina DJ, Liu C-G (2008). Role of MicroRNA miR-27a and miR-451 in the regulation of MDR1/P-glycoprotein expression in human cancer cells. Biochem Pharmacol.

[57] Zhou J-H, Zhou Q-Z, Yang J-K, Lyu X-M, Bian J, Guo W-B, et al. MicroRNA-27a-mediated repression of cysteine-rich secretory protein 2 translation in asthenoteratozoospermic patients. 2016; available from: www.ajandrology.com/preprintarticle.asp?id=185001. 10.4103/1008-682X.185001 PMC5566855 27517483

[58] Safarinejad MR, Shafiei N, Safarinejad S (2010). Association of polymorphisms in the estrogen receptors alpha, and beta (ESR1, ESR2) with the occurrence of male infertility and semen parameters. J Steroid Biochem Mol Biol.

[59] Carreau S, Genissel C, Bilinska B, Levallet J (1999). Sources of oestrogen in the testis and reproductive tract of the male. Int J Androl.

[60] Abhari A, Zarghami N, Shahnazi V, Barzegar A, Farzadi L, Karami H (2014). Significance of microRNA targeted estrogen receptor in male fertility. Iran J Basic Med Sci.

[61] Li Z, Zheng Z, Ruan J, Li Z, Zhuang X, Tzeng C-M (2016). Integrated analysis miRNA and mRNA profiling in patients with severe oligozoospermia reveals miR-34c-3p downregulates PLCXD3 expression. Oncotarget.

[62] Tang D, Huang Y, Liu W, Zhang X (2016). Up-regulation of microRNA-210 is associated with spermatogenesis by targeting IGF2 in male infertility. Med Sci Monit.

[63] Krawetz SA, Kruger A, Lalancette C, Tagett R, Anton E, Draghici S (2011). A survey of small RNAs in human sperm. Hum Reprod.

[64] Cui L, Fang L, Shi B, Qiu S, Ye Y (2015). Spermatozoa micro ribonucleic acid–34c level is correlated with intracytoplasmic sperm injection outcomes. Fertil Steril.

[65] Girard A, Sachidanandam R, Hannon GJ, Carmell MA. A germline-specific class of small RNAs binds mammalian Piwi proteins. Nature [Internet]. 2006 [cited 2017 Feb 20]; Available from: http://www.nature.com/doifinder/10.1038/nature04917 10.1038/nature04917 16751776

[66] Seto AG, Kingston RE, Lau NC (2007). The Coming of age for piwi proteins. Mol Cell.

[67] Siomi MC, Sato K, Pezic D, Aravin AA (2011). PIWI-interacting small RNAs: the vanguard of genome defence. Nat Rev Mol Cell Biol.

[68] He Z, Kokkinaki M, Pant D, Gallicano GI, Dym M (2009). Small RNA molecules in the regulation of spermatogenesis. Reproduction.

[69] Yang Q, Hua J, Wang L, Xu B, Zhang H, Ye N (2013). MicroRNA and piRNA profiles in normal human testis detected by next generation sequencing. PLoS ONE.

[70] Gou L-T, Dai P, Liu M-F (2014). Small noncoding RNAs and male infertility: Small ncRNAs and male infertility. Wiley Interdiscip Rev RNA.

[71] Hilz S, Modzelewski AJ, Cohen PE, Grimson A (2016). The roles of microRNAs and siRNAs in mammalian spermatogenesis. Development.

[72] Dong TT, Yu Q, Qing XR, Ma XL, Dong WW, Shi J (2016). Potential confounding factors in measurement of specific cell-free seminal mRNAs and microRNAs derived from human reproductive organs. Androl.

